# SKP alleviates the ferroptosis in diabetic kidney disease through suppression of HIF-1α/HO-1 pathway based on network pharmacology analysis and experimental validation

**DOI:** 10.1186/s13020-024-00901-5

**Published:** 2024-02-25

**Authors:** Yangtian Yan, Ningning Yuan, Yuchi Chen, Yun Ma, Ali Chen, Fujing Wang, Shihua Yan, Zhuo’en He, Jinyue He, Chi Zhang, Hao Wang, Mingqing Wang, Jianxin Diao, Wei Xiao

**Affiliations:** 1 School of Traditional Chinese Medicine, Southern Medical University, Guangzhou, Guangdong China; 2 Key Laboratory of Glucolipid Metabolic Disorder, Ministry of Education, Guangdong Pharmaceutical University, Guangzhou, Guangdong China; 3 Clinical Pharmacy Center, Nanfang Hospital, Southern Medical University, Guangzhou, Guangdong China; 4 Center for Drug Research and Development, Guangdong Provincial Key Laboratory of Advanced Drug Delivery System, Guangdong Pharmaceutical University, Guangzhou, Guangdong China

**Keywords:** Diabetic kidney disease (DKD), Shenkang pills (SKP), Ferroptosis, Network pharmacology, HIF-1*α*/HO-1 signaling pathway

## Abstract

**Background:**

Diabetic kidney disease (DKD) represents a microvascular complication of diabetes mellitus. Shenkang Pills (SKP), a traditional Chinese medicine formula, has been widely used in the treatment of DKD and has obvious antioxidant effect. Ferroptosis, a novel mode of cell death due to iron overload, has been shown to be associated with DKD. Nevertheless, the precise effects and underlying mechanisms of SKP on ferroptosis in diabetic kidney disease remain unclear.

**Methods:**

The active components of SKP were retrieved from the Traditional Chinese Medicine Systems Pharmacology (TCMSP) database. Protein–protein interaction (PPI) network and Herb-ingredient-targets gene network were constructed using Cytoscape. Gene Ontology (GO) and Kyoto Encyclopedia of Genes and Genomes (KEGG) pathway enrichment analyses were conducted utilizing the Metascape system database. Additionally, an in vivo model of DKD induced by Streptozotocin (STZ) was established to further investigate and validate the possible mechanisms underlying the effectiveness of SKP.

**Results:**

We retrieved 56 compounds and identified 223 targets of SKP through the TCMSP database. Key targets were ascertained using PPI network analysis. By constructing a Herb-Ingredient-Targets gene network, we isolated the primary active components in SKP that potentially counteract ferroptosis in diabetic kidney disease. KEGG pathway enrichment analysis suggested that SKP has the potential to alleviate ferroptosis through HIF signaling pathway, thereby mitigating renal injury in DKD. In animal experiments, fasting blood glucose, 24 h urine protein, urea nitrogen and serum creatine were measured. The results showed that SKP could improve DKD. Results from animal experiments were also confirmed the efficacy of SKP in alleviating renal fibrosis, oxidative stress and ferroptosis in DKD mice. These effects were accompanied by the significant reductions in renal tissue expression of HIF-1α and HO-1 proteins. The mRNA and immunohistochemistry results were the same as above.

**Conclusions:**

SKP potentially mitigating renal injury in DKD by subduing ferroptosis through the intricacies of the HIF-1α/HO-1 signaling pathway.

**Supplementary Information:**

The online version contains supplementary material available at 10.1186/s13020-024-00901-5.

## Introduction

Last several years, the global prevalence of Diabetes mellitus (DM) has been on the rise, paralleled by a gradual increase in diabetes-related diabetic kidney disease (DKD) [1]. DKD, a severe microvascular complication of DM, presents clinically with elevated blood glucose, proteinuria and diminished glomerular filtration rate [2]. Notably, DKD stands as the foremost cause of end-stage renal disease(ESRD) [3]. Despite the escalating burden of DKD, clinical practice faces limitations in the availability of effective Western drugs. Currently, Angiotensin II receptor blocker or Angiotensin-converting enzyme inhibitor are commonly employed to impede the renin-angiotensin-aldosterone system to slow down the progression of DKD [4].However, conventional renin-angiotensin-aldosterone system inhibitors carry notable side effects, including the risk of hyperkalemia [5]. Consequently, there exists an imperative to identify novel therapeutic targets and cultivate innovative drugs to address this pressing healthcare challenge.

Traditional Chinese medicine (TCM) has been widely used in the prevention and treatment of DKD, offering notable efficacy and minimal adverse reactions [6, 7]. A case is the Shenkang Pill (SKP), a hospital prescription refined from an effective traditional Chinese medicine formula specifically tailored for DKD based on traditional Chinese medicine principles. SKP, comprising of Astragalus membranaceus, Eucommia ulmoides, Euryale ferox Salisb, Leonurus japonicus Houtt, Rosa laevigata Michx, Corn Silk, Hawthorn and Houttuynia cordata, has garnered extensive clinical use over the years. We previously identified compounds in SKP by using the method of LC-Q-TOF-MS, and quantified total polysaccharides, organic acids, total flavonoids, total saponins, total protein. Major categories in SKP include polysaccharides, organic acids, flavonoids, saponins, and purine derivatives etc. Our prior research also demonstrated SKP’s efficacy in mitigating renal injury and reducing proteinuria in db/db mice [8]. SKP also has antioxidant properties. However, given its intricate composition, unravelling the mechanism behind SKP’s therapeutic impact on DKD warrants further exploration.

Different from programmed cell death mechanisms such as apoptosis, pyroptosis and autophagy, ferroptosis is a new mode of cell death that is iron-dependent [9, 10]. Characterized by iron-mediated reactive oxygen species (ROS) oxidation of unsaturated fatty acids, ferroptosis results in lipid peroxide production, causing cell membrane damage and loss of membrane integrity [11, 12]. Growing evidence underscores the pivotal role of ferroptosis in the development of DKD. ROS are small molecular substances mainly derived from the respiratory chain of the inner mitochondrial membrane [13]. The ferroptosis process will produce a large amount of ROS, and renal tubules are one of the tissues with the highest content of mitochondria [14], which is more prone to oxidative stress and damage. Prior studies indicate that ferroptosis is the primary mode of death for renal tubular cells [15], exacerbating tubular injury and interstitial fibrosis in DKD [16]. While most DKD researches traditionally focuses on glomeruli, the clinical importance of renal tubules is gradually being recognized. In addition, the tubulointerstitial compartment accounts for 90% of the renal parenchymal mass, which also provides support that renal tubular injury plays an important role in DKD progression [17].

In our previous work, we established the efficacy of SKP in ameliorating renal tubular injury in DKD model [8]. However, the specific mechanisms through which SKP exerts its therapeutic effects, particularly in relation to ferroptosis, were not fully elucidated. This introduction sets the stage for discussing our hypothesis that SKP mitigates renal tubular injury through inhibition of ferroptosis, our subsequent research employs network pharmacology to explore the relationship between DKD, SKP and ferroptosis, emphasizing a multi-target, multi-component therapeutic approach [18].

## Materials and methods

### Materials and dose design of SKP

SKP is composed of 8 traditional Chinese herbs: Astragalus membranaceus, Eucommia ulmoides, Euryale ferox Salisb, Leonurus japonicus Houtt, Rosa laevigata Michx, Corn Silk, Hawthorn, Houttuynia cordata.

The preparation of SKP was conducted in our laboratory as follows: Astragalus membranaceus 60 g, Eucommia ulmoides 15 g, Euryale ferox Salisb 30 g, Leonurus japonicus Houtt 15 g, Rosa laevigata Michx 30 g, Corn Silk 30 g, Hawthorn 30 g and Houttuynia cordata 30 g. All these herbs were sourced from the Nanfang Hospital Affiliated to Southern Medical University (Guangzhou, China). After the above herbs were mixed in corresponding proportions and decocted twice with water (1680 mL) for 1 h, then the supernatants were combined. The mixture was concentrated into 0.91 g/ml and 0.455 g/ml and stored at – 20 °C for use in animal experiments.

#### Reagents

The Urine protein test kit (C035-2-1), Urea Assay kit (C013-2-1), Creatinin Assay kit (C011-2-1), Malondialdehyde (MDA) assay kit (A003-1-2), Reduced glutathione (GSH) assay kit (A006-2-1), tissue iron assay kit (A039-2-1) were all obtained from Jiancheng Bioengineering Institute (Nanjing, China). The Dihydroethidium (S0063) was obtained from Beyotime (Jiangsu, China). Streptozotocin (STZ) (S0130-1g) was acquired from Sigma-Aldrich (St. Louis, Missouri, USA). Ferrous Ion Content Assay Kit (BC5415) was acquired from Solarbio (Beijing, China). qPCR SYBR Green Master Mix (11198ES08) was purchased from Yeasen Biotechnology (Shanghai, China). GPX4 antibody(67763-1-Ig), GAPDH antibody (60004-1-Ig) and α-SMA antibody (67735-1-Ig) were purchased from Proteintech (Wuhan, China). HIF-1α antibody (340462), HO-1 antibody (R24541) and Fibronectin antibody (250073) were purchased from ZEN-BIOSCIENCE (Chengdu, China). Vimentin antibody (AF7013) and β-actin antibody (AF7018) were purchased from Affinity Biosciences (Jiangsu, China). HRP AffiniPure Goat Anti-Mouse IgG (H+L) (FDM007) and HRP AffiniPure Goat Anti-Rabbit IgG (H+L) (FDR007) were obtained from Fude Biological Technology (Hangzhou, China).

#### Animals and experimental design

Studies suggest that C57BL/6 mice share many similar features with human metabolic syndrome, including insulin resistance and glucose intolerance etc. [19]. Hence, we chose C57BL/6 mice to establish a DKD model. Male C57BL/6 mice aged 4 weeks were purchased from the Experimental Animal Center of Southern Medical University. Experimental animals were kept in an environment maintained at a temperature (22 °C), lighting (12 h light/dark cycle). The mice were allowed to eat and drink freely.

After one week of adaptive feeding, mice were randomly assigned into 5 groups before the experiment: control group (n = 6), model group (n = 6), low-dose SKP group (SKPL) (n = 6), high-dose SKP group (SKPH) (n = 6), and Valsartan group (VAL) (n = 6).

Except for the control group, the other mice were fed with a high-fat diet (HFD). After 4 weeks, STZ (40 mg/kg, i.p, dissolved in 0.1 M citrate-sodium citrate buffer) was injected intraperitoneally for 5 consecutive days. The control group only received the same dose of citrate-sodium citrate buffer. After STZ injection, the mice developed polydipsia, polyphagia, polyuria and a gradual decrease in body weight. Fasting blood glucose (FBG) of the mice were measured after 3 days, and a concentration ≥ 11.1 mmol/l was considered the standard for DKD model.

With a total formulation weight of 240 g and normal adult calculation based on a human (60 kg), the dose for adults is 4 g/d per unit weight. Using the conversion coefficient of 9.1 [20], this translates to a mouse dose of 36.4 g/kg. The lower dose is half of this amount. For Valsartan, the adult daily dose is 80 mg, equivalent to 1.3 mg/kg. Applying the same conversion, the mouse daily dose is 11.83 mg/kg. The mice were then given SKP (36.4 g/kg, i.g, once daily) in the SKPH group, SKP (18.2 g/kg, i.g, once daily) in the SKPL group, and Valsartan (11.83 mg/kg, i.g, once daily), a positive control drug, in the VAL group. Additionally, mice in the model group were treated with 0.5 % CMC-Na solution in equal volume for 7 weeks. Mice were sacrificed after 7 weeks of treatment. Then the tissues were snap-frozen in liquid nitrogen and stored at -80°C until experiments were performed. All the experimental protocols were approved by the Institutional Animal Care and Use Committee for Southern Medical University. (Ethical number: L2022047; license number: SCXK(Yue)2021-0041, SYXK(Yue)2021-0167).

#### Biochemical analyses

Glucose levels in the tail vein blood of the mice were measured with a glucometer. Metabolic cages were used to collect urine from animals, and the urinary protein con- centration was measured by a Urine protein test kit. The upper layer of serum was taken to detect serum related indicators as per the instructions of the test kit. This included assessing Blood Urea Nitrogen (BUN) and Serum creatinine (Scr) for renal function.

#### Assessment of GSH, MDA, ROS, Fe^2+^and iron concentration

Kidney tissues were added to a ninefold volume of normal saline and harvested for tissue homogenization. The supernatants of each group were collected, and the content was detected with Malondialdehyde (MDA) assay kit, Reduced glutathione (GSH) assay kit, tissue iron assay kit, Ferrous Ion Content Assay Kit. The level of intracellular ROS in renal tissues were detected by dihydroethidium.

#### Network pharmacology analysis

##### Screening of active ingredients in SKP

To identify the active ingredients in SKP, we accessed the Traditional Chinese Medicine Systems Pharmacology database (TCMSP, https://tcmsp-e.com/tcmsp.php). The chemical composition and corresponding targets of SKP were retrieved through TCMSP by searching the keywords of “Astragalus membranaceus” “Eucommia ulmoides” “Euryale ferox Salisb” “Leonurus japonicus Hout” “Rosa laevigata Michx” “Corn Silk” “Hawthorn” and “Houttuynia cordata”. Our selection criteria included Oral Bioavailability (OB) value of ≥ 30% and Drug-likeness (DL) value of ≥ 0.18.

##### Collection of targets

To obtain relevant targets associated with Diabetic Kidney Disease and ferroptosis, we utilized the GeneCards databases. Subsequently, we standardized the names of these targets using the UniProt database (UniProt Consortium, 2018).

##### Venn diagram and PPI network

The targets of SKP, DKD and ferroptosis were screened and visualized with Venn diagrams. In the protein–protein interaction (PPI) network, nodes represent genes, proteins or molecules, while the lines between nodes represent associations. The intersection of SKP targets, DKD targets and ferroptosis targets were taken to obtain the target of ferroptosis induced by SKP in DKD. The targets were entered into the STRING database to construct the PPI network, and these data were analyzed using Cytoscape (version 3.9.1) to build a visualized compound-target network.

##### Component-target-disease network

To visualize the interactions between the active components of each herb in SKP and their potential targets, data were imported into Excel and then used to construct a comprehensive component-target-disease network. This network was created and visualized using Cytoscape (version 3.9.1), providing a clear representation of how these components and targets are interconnected in the context of disease.

##### GO and KEGG enrichment analysis

The shared targets of SKP, DKD and ferroptosis were imported into the Metascape system database (http://metascape.org) for GO and KEGG enrichment analysis. GO enrichment analysis included cellular component (CC), molecular function (MF) and biological process (BP).

The top 20 results of KEGG enrichment analysis were imported into the bioinformatics website (https://www.bioinformatics.com.cn/), and the data were presented through informative bubble plots. Used P<0.05 as the threshold to determine statistically significant results.

##### Molecular docking

The structures of the compounds obtained from SKP are all from the PubChem database. These structures were then converted into a three-dimensional (3D) structure and their energy were minimized using Chem 3D. Protein Data Bank (PDB; https://www.rcsb.org/) was used to obtain crystal structures of the proteins. Subsequently molecular docking was performed using AutoDucktools1.5.7.

##### Histological evaluation

Kidneys were fixed in 4% paraformaldehyde buffer, dehydrated in ethanol, embedded in paraffin, and made into 4 µm sections. For histological evaluation, hematoxylin and eosin (H&E) and periodic acid Schiff (PAS) staining were conducted. Prussian blue staining was used to detect Iron deposition levels. Additionally, Masson’s staining and Sirius red staining were employed to evaluate the extent of renal fibrosis.

##### Immunohistochemistry

Immunohistochemistry (IHC) was performed on kidney tissue sections to assess for the expression of GPX4, HIF-1α and HO-1. The tissue sections underwent deparaffinization, hydration and antigen retrieved. Subsequently, they were incubated with 5% goat serum, primary antibodies against GPX4 (dilution 1:200), HIF-1α (dilution 1:50), HO-1 (dilution 1:50) antibodies at 4 °C overnight. Afterward, the sections were treated with secondary antibody (dilution 1:1000) at 37 °C for 1 h. The sections were then stained with DAB and counterstained with hematoxylin before sealing with neutral resin.

##### Western blotting

RIPA lysate buffer and pmsf were used to extract proteins from kidney tissue. The lysates were subsequently centrifuged at 12,000*g* for 20 min, and the protein concentrations were measured with the use of the BCA assay. Protein aliquots (30 µg) were separated on 10–12% SDS-PAGE gels, followed by transfer to PVDF membrane. The membranes were blocked in TBST solution containing 5% skim milk. Afterwards, membranes were incubated with primary antibodies in following dilutions:GPX4 (dilution 1:1000), HIF-1α (dilution 1:1000), HO-1 (dilution 1:1000), Fibronectin (dilution 1:1000), α-SMA (dilution 1:1000), Vimentin (dilution 1:1000), GAPDH (dilution 1:1000), β-actin (dilution 1:1000). Blots were incubated at 4 ◦C overnight, the membranes were treated with HRP AffiniPure Goat.

Anti-Rabbit IgG (H+L) secondary antibodies (dilution 1:10,000), HRP AffiniPure Goat Anti-Mouse IgG (H+L) secondary antibodies (dilution 1:1000) for 2 h at 4 °C. In the final steps of the process, the electro-chemiluminescence signals of the protein bands were detected using enhanced chemiluminescence reagent after washing. Protein band intensities were quantified using ImageJ software.

### Quantitative real-time polymerase chain reaction (qRT-PCR)

Total RNA was initially extracted from kidney tissues using Trizol and phenol–chloroform extraction. PCR amplification was then performed, Finally, 2^−ΔΔCT^ method was used to analyze the results.

### Statistical analysis

In statistical analysis, we used data values as mean standard deviation. P < 0.05 was considered statistically significant. Differences between two groups were analyzed using Student’s t-test, and differences between more than two groups were analyzed using one-way analysis of variance. GraphPad Prism 8 was used for data analysis.

## Results

### Therapeutic effects of SKP on DKD mice

**Fig. 1:**
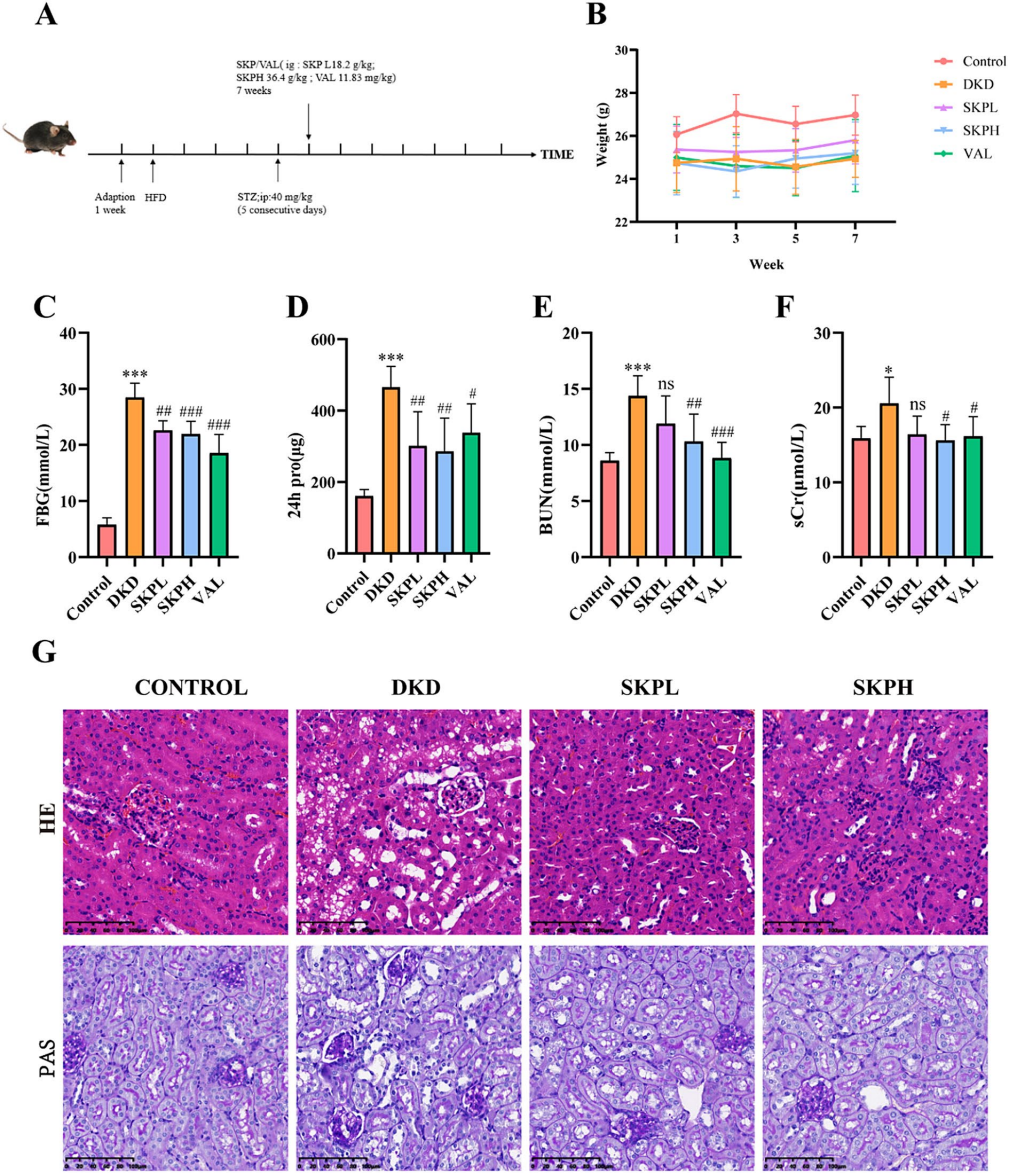
SKP alleviates kidney injury in DKD mice. **A** After 1 week of adaptive feeding, the mice were fed with HFD for 4 weeks and then intraperitoneally injected with STZ 40 mg/kg/ day for 5 consecutive days. After the model was successfully established, the drug groups were given oral feeding, and the model group (n = 6) was given the same volume of normal saline. The treatment lasted for 7 weeks. **B**–**G** SKP and Val alleviated kidney damage and fasting blood glucose (FBG) in DKD mice. **B** Changes in body weight of mice in each group during drug administration. **C** FBG level. **D** 24-h urine protein. **E** BUN levels. **F** Serum creatinine level. **G** H&E staining and PAS staining of kidney tissues of mice (400 × magnification, bar = 100 µm). *P < 0.05, **P < 0.01, and ***P < 0.001vs. control group. ^#^P < 0.05, ^##^P < 0.01, and ^###^P < 0.001 vs. DKD group, *ns* no significance

To investigate the therapeutic effect of SKP on DKD, a mouse model of diabetic kidney disease was established (Fig. 1A). The body weights of mice were dynamically observed every 2 weeks from the beginning of drug administration. Compared with control group, the body weights of mice in the DKD, SKPL, SKPH and VAL groups increased, but there was no statistical significance (Fig. 1B). As shown in Fig. 1C, FBG was significantly increased in the DKD group, however, SKP and valsartan could reduce FBG levels, indicating their potential in controlling hyperglycemia associated with diabetic kidney disease. We next performed renal function tests, the results showed that 24h urine protein levels, urea nitrogen and serum creatine increased significantly in the DKD group with statistically significant results, but decreased after the SKP intervention treatment. Moreover, the SKPH group had a greater improvement than the SKPL group (Fig. 1D–F).

HE staining revealed that in the control group, the morphologies of glomeruli, tubules and renal interstitium were within the normal range. In contrast, mild glomerular basement membrane thickening, tubular vacuolar degeneration and renal interstitial edema were observed in the DKD group. PAS staining further confirmed significant increases in glycogen and glomerular basement membrane thickness in the DKD group, with these changes showing improvement following SKP treatment. Notably, kidney damage was evident in the DKD group compared to the control group, but these manifestations were mitigated to varying degrees after SKP intervention (Fig. 1G). In summary, our findings demonstrate that SKP treatment not only effectively lowers fasting glucose levels but also significantly ameliorates kidney damage in a DKD mouse model. This highlights the potential of SKP as a therapeutic agent in managing diabetic kidney disease, with its effects being more pronounced at higher dosages.

### SKP reduced the fibrosis in DKD mice

**Fig. 2:**
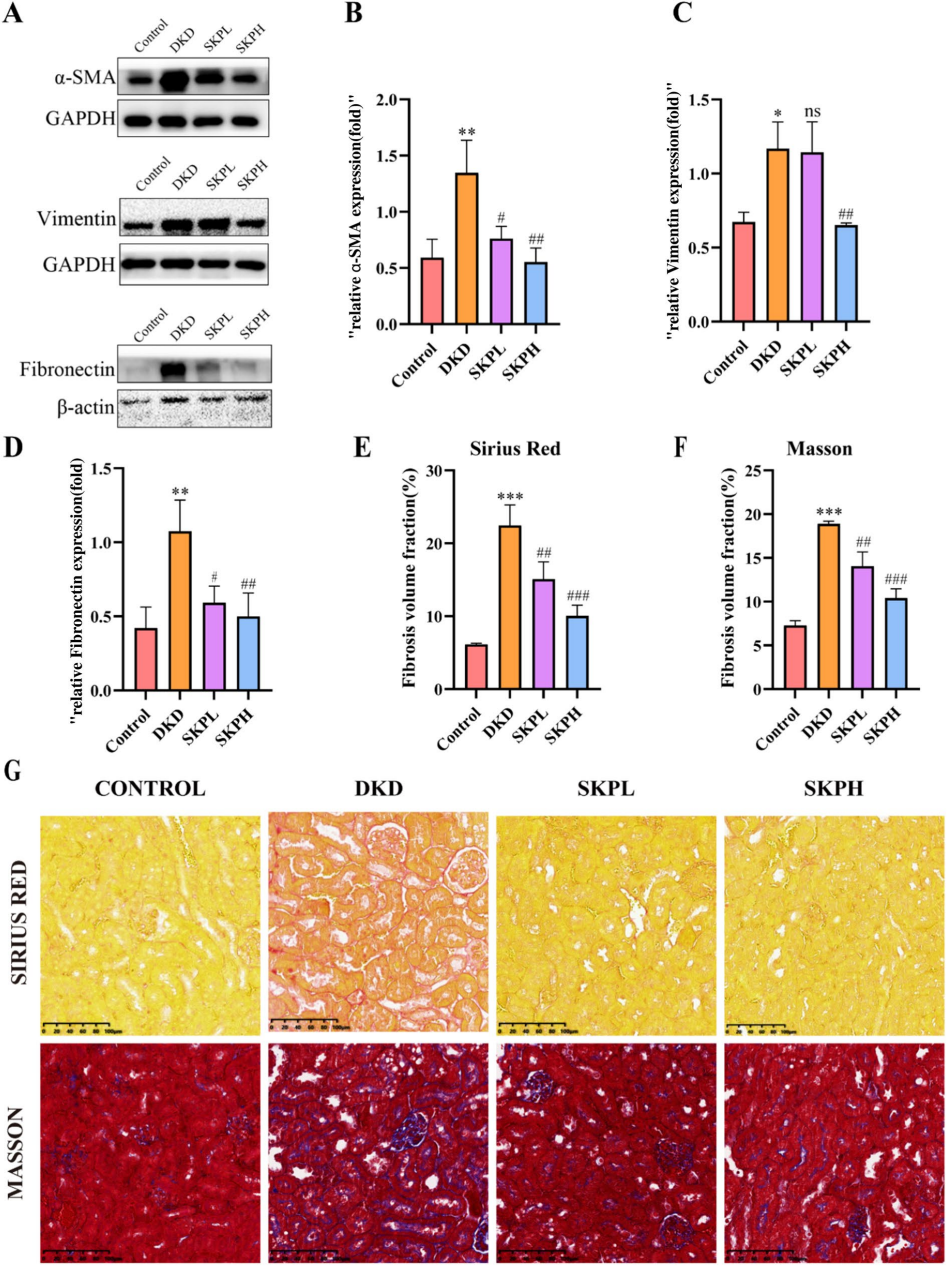
SKP alleviated renal fibrosis in DKD mice. **A** Western blot was performed to determine the expression of α-SMA Fibronectin and Vimentin in kidney tissues of each group. **B**–**D** The quantitative analysis for Western blotting results of α-SMA, Fibronectin and Vimentin. **E**, **F** Quantification of renal tissue fibrosis in mice. **G** Masson and Sirius red staining of kidney tissues of mice (400 × magnification, bar = 100 µm). *P < 0.05, **P < 0.01, and ***P < 0.001vs. control group. ^#^P < 0.05, ^##^P < 0.01, and ^###^P < 0.001 vs. DKD group, *ns* no significance

Indepth analysis conducted through Western blot techniques has provided compelling evidence. The relative expression levels of fibrogenesis-related proteins α-SMA, Fibronectin and Vimentin within the renal tissues of mice afflicted with DKD were found to be markedly elevated (Fig. 2A–D). Yet, notably, the administration of SKP was associated with a reduction of these protein expression level, highlighting the therapeutic potential of SKP in addressing the molecular alterations induced by diabetic kidney disease.

As exhibited in Fig. 2G, Masson and Sirius Red staining are extensively employed to meticulously examine the collagen fiber distribution within tissues [21]. As elucidated by the results obtained through these staining techniques, diabetes has been observed to substantially augment fibrosis within the renal tissues of mice, underscoring the detrimental impact of this condition.

Distribution statistics have yielded insightful data, revealing that treatment with varying dosages of SKP has the capacity to mitigate the area of renal fibrosis, demonstrating a correlation between the dose administered and the degree of reduction observed (Fig. 2E, F).

### Effects of SKP on ferroptosis in the kidneys of DKD mice

**Fig. 3:**
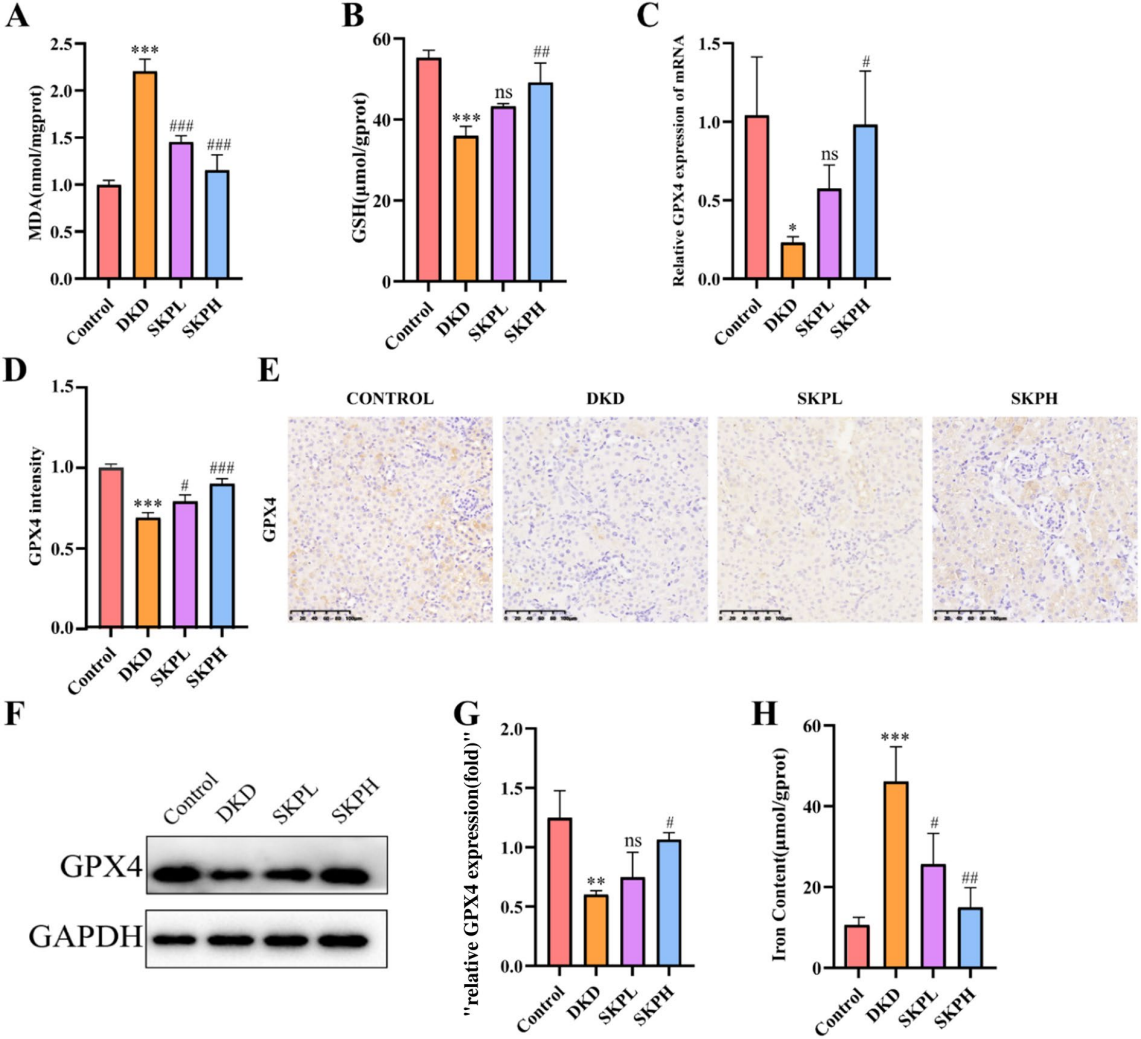
SKP reduced ferroptosis of kidney tissues in DKD mice. **A**, **B** The levels of MDA (Malonaldehyde) and GSH(Glutathione) in kidney tissues of mice. **C** The mRNA levels of GPX4 in the kidneys of each group. **D** The quantitative analysis for immunohistochemical staining assays of GPX4. **E** Immunohistochemical staining assays of GPX4 in kidney tissues. (400 × magnification, bar = 100 µm). **F**, **G** Western blot for GPX4 in kidney tissues of each group and the quantitative analysis for Western blotting results of GPX4. **H** The levels of Iron Content in kidney tissues. *P < 0.05, **P < 0.01, and ***P < 0.001 vs. control group. ^#^P < 0.05, ^##^P < 0.01, and ^###^P < 0.001 vs. DKD group, *ns* no significance

To evaluate whether SKP could alleviate renal ferroptosis induced by DKD, we conducted analysis of GSH, MDA levels (Fig. 3A, B). The results depicted in the figures revealed a substantial increase in the lipid peroxidative product MDA in DKD mice when compared to the control group. Conversely, the GSH content was significantly decreased in DKD group. Following SKP intervention, the aforementioned indexes in DKD mice exhibited varying degrees of improvement.

GPX4 was identified as a pivotal regulator of ferroptosis. Notably, SKP treatment led to a significant upregulation of GPX4 mRNA expression (Fig. 3C). The result showed a consistent trend with IHC. Quantitative analysis of immunohistochemical staining was performed using Image J (Fig. 3D, E). We observed the decrease in GPX4 protein (Fig. 3F, G) in DKD mice when contrasted with normal mice which was alleviated by SKP treatment. As exhibited in Fig. 3H, the levels of Iron Content in DKD mice increased significantly.

### Network pharmacology analysis

####  Disease targets

**Fig. 4:**
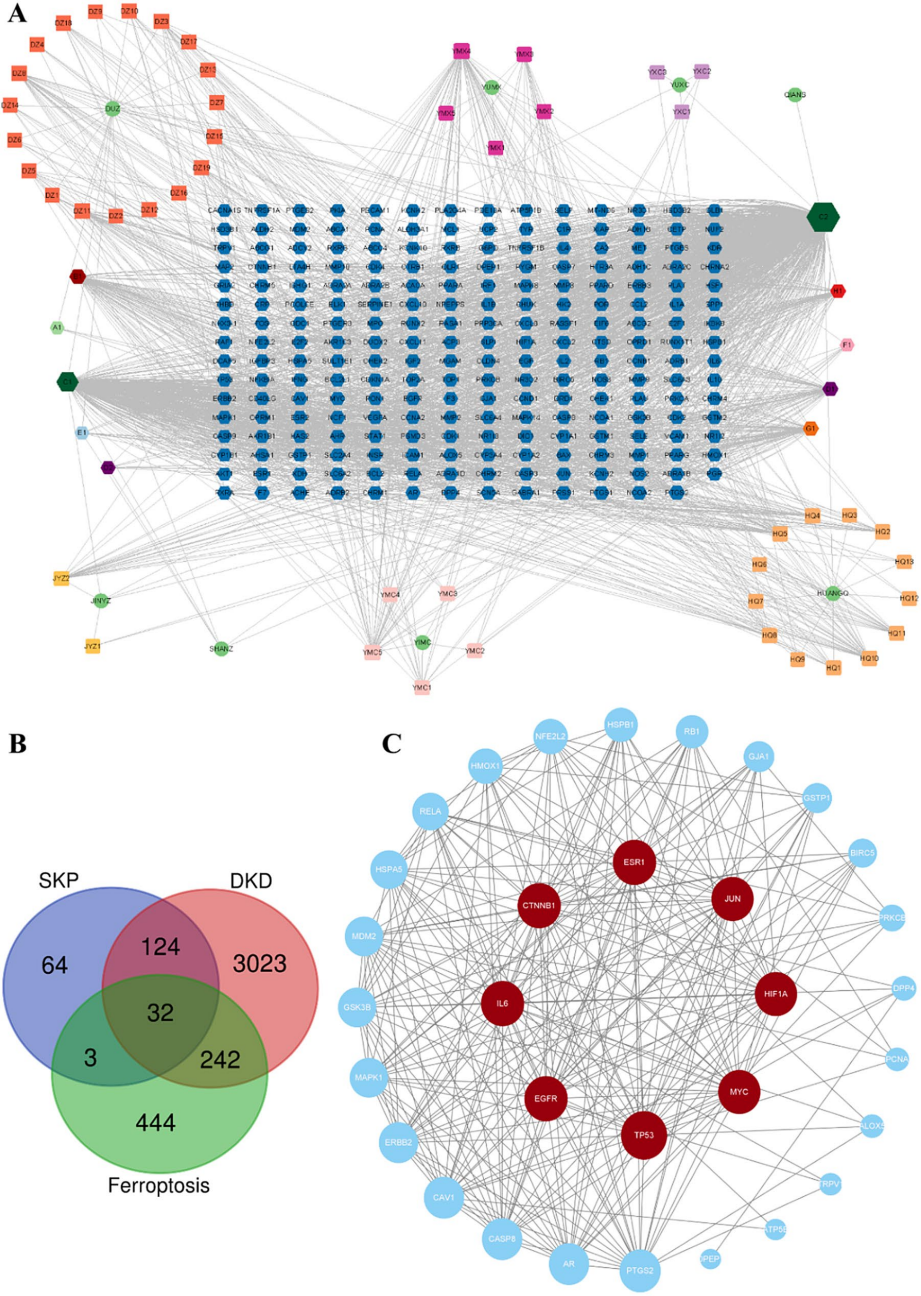
Network pharmacology analysis. **A** The Herb-ingredient-targets gene network. Green circle nodes represented herbs in SKP, rectangles represented compounds in SKP, and blue hexagonal nodes represented the intersectant targets. A1 to H1 represented shared components of two or more herbs in SKP, the details were listed in Additional file 1: Table S1. **B** protein–protein interaction (PPI) network of the putative targets. The red circle nodes in the middle represent the eight targets with the highest degree values. **C** Venn diagram of the targets of SKP, DKD and ferroptosis

After removing duplicates in the database, a total of 223 active SKP compounds, 3421 targets of DKD and 721 ferroptosis targets were obtained. The three targets were combined to obtain a total of 32 common targets. A Venn diagram was used to visually represent the overlapping targets between components and diseases (Fig.4B).

#### Construction of PPI network and Herb-ingredient-targets gene network

Subsequently, we constructed a Protein-protein interaction (PPI) network (Fig. 4C) and Herb-ingredient-targets gene network (Fig. 4A). The 32 targets were imported into the STRING database (https://stringdb.org) to construct the PPI network. SKP active ingredients and targets were linked to elucidate their interactions. The results were visualized using Cytoscape3.9.1 software. Notably, the top 8 important targets, determined by degree value, included TP53, IL6, EGFR, CTNNB1, ESR1, JUN, MYC, and HIF1A (Table 1). At the same time, Cytoscape3.9.1 was used to construct the Herb-ingredient-targets gene network. Among the active compounds, those with the highest degree values were quercetin, kaempferol, isorhamnetin, beta-sitosterol, luteolin, stigmasterol and beta-carotene. These components were considered to be the main active components of SKP in the treatment of ferroptosis in DKD.

**Table 1:** Summary of the top 8 core targets of SKP in treatment of DKD in PPI network

Number	Target	Degree centrality
1	TP53	54
2	EGFR	48
3	IL6	48
4	CTNNB1	48
5	ESR1	48
6	JUN	46
7	HIF1A	46
8	MYC	46

###  Results of GO and KEGG analyses

**Fig. 5:**
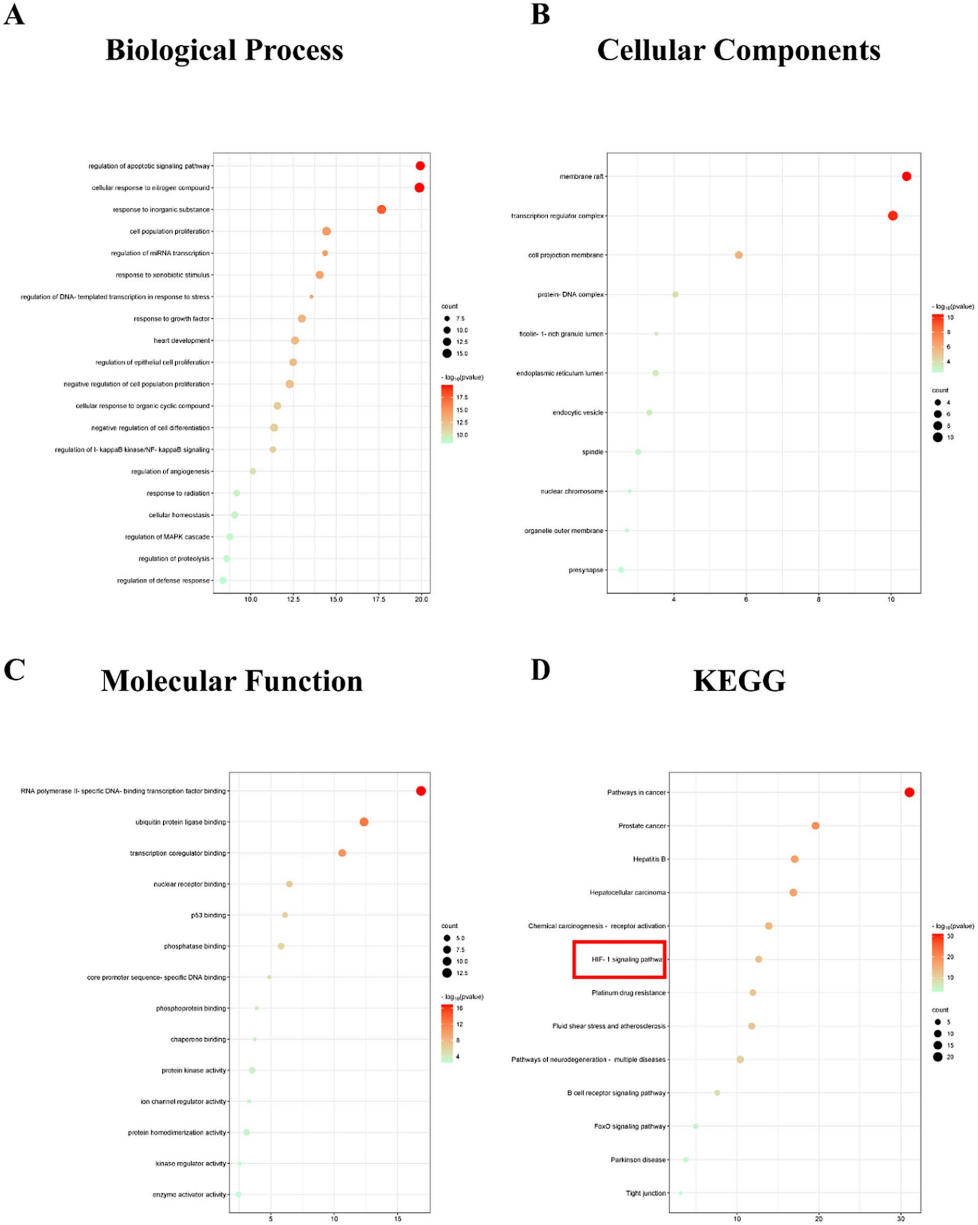
Diagrams of GO and KEGG enrichment analyses. **A**–**D** The color of the dot represented the − log10 (Pvalue) value, the size represented the gene count

To investigate the therapeutic potential of SKP in mitigating ferroptosis within the context of DKD, GO and KEGG pathway enrichment analyses were performed using Matescape. GO enrichment analysis was divided into BP, CC and MF. The main BP terms involved regulation of apoptotic signaling pathway, cellular response to nitrogen compound and response to inorganic substance (Fig. 5A). The main CC terms involved membrane raft, transcription regulator complex and cell projection membrane (Fig. 5B). RNA polymerase II-specific DNA-binding transcription factor binding and ubiquitin protein ligase was included in MF binding (Fig. 5C). For KEGG enrichment analysis, our results indicated that the target genes were primarily associated with cancer pathway and HIF signaling pathway (Fig. 5D). These insights provide valuable information on the molecular mechanisms underlying SKP’s potential therapeutic effect on ferroptosis in DKD.

### Molecular docking

**Fig. 6:**
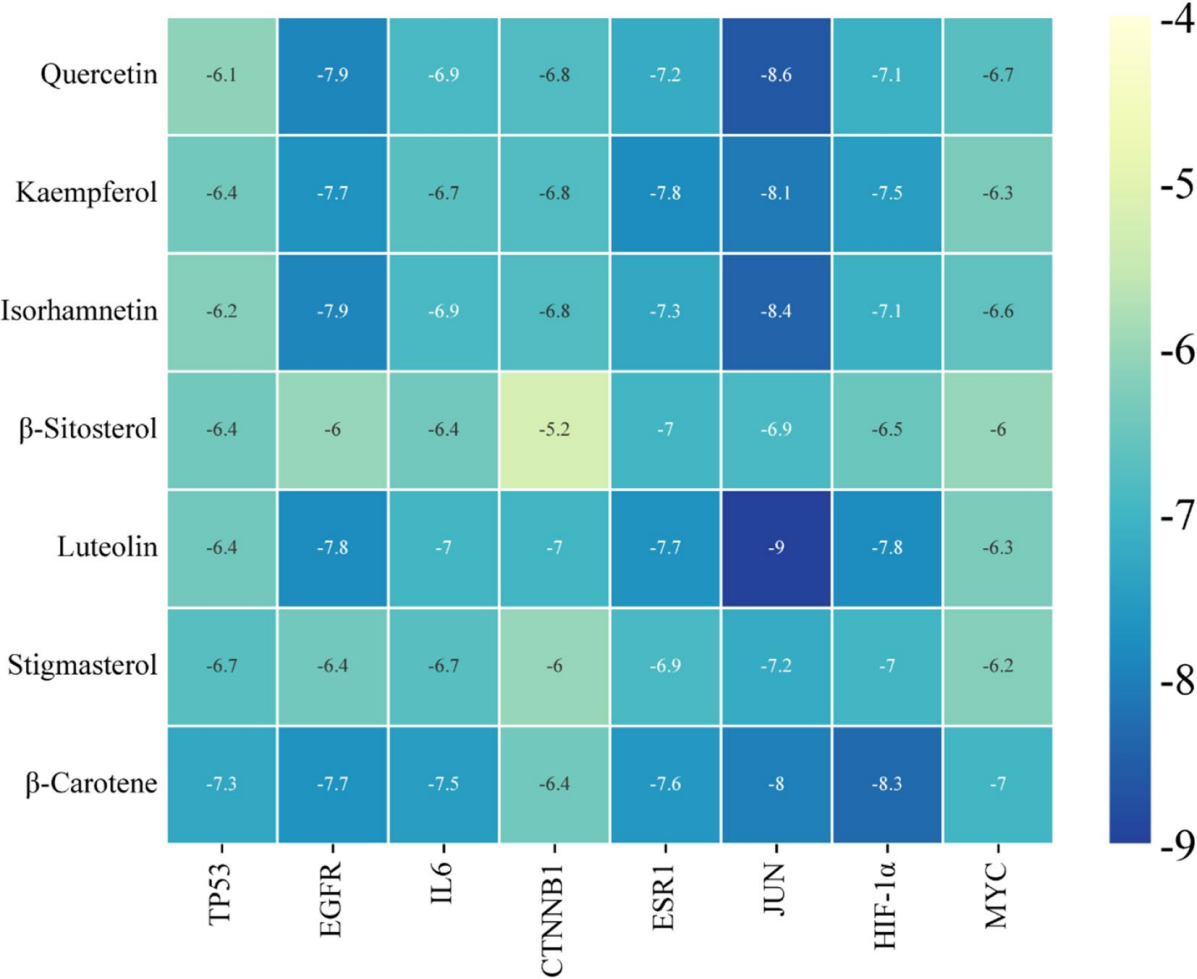
Heatmap of binding between main components and targets. The darker the color, the better the binding

As shown in Fig. 6, the top eight important targets (TP53, IL6, EGFR, CTNNB1, ESR1, JUN, MYC and HIF1A) were used for molecular docking with the main active compounds of SKP, and the results were visualized as a heatmap. An affinity of less than -7.0 kcal/mol indicates a high binding capacity [22]. The results showed that HIF-1α was well bound to the active ingredients.

### SKP inhibited the HIF-1α/HO-1 signaling pathway

**Fig. 7:**
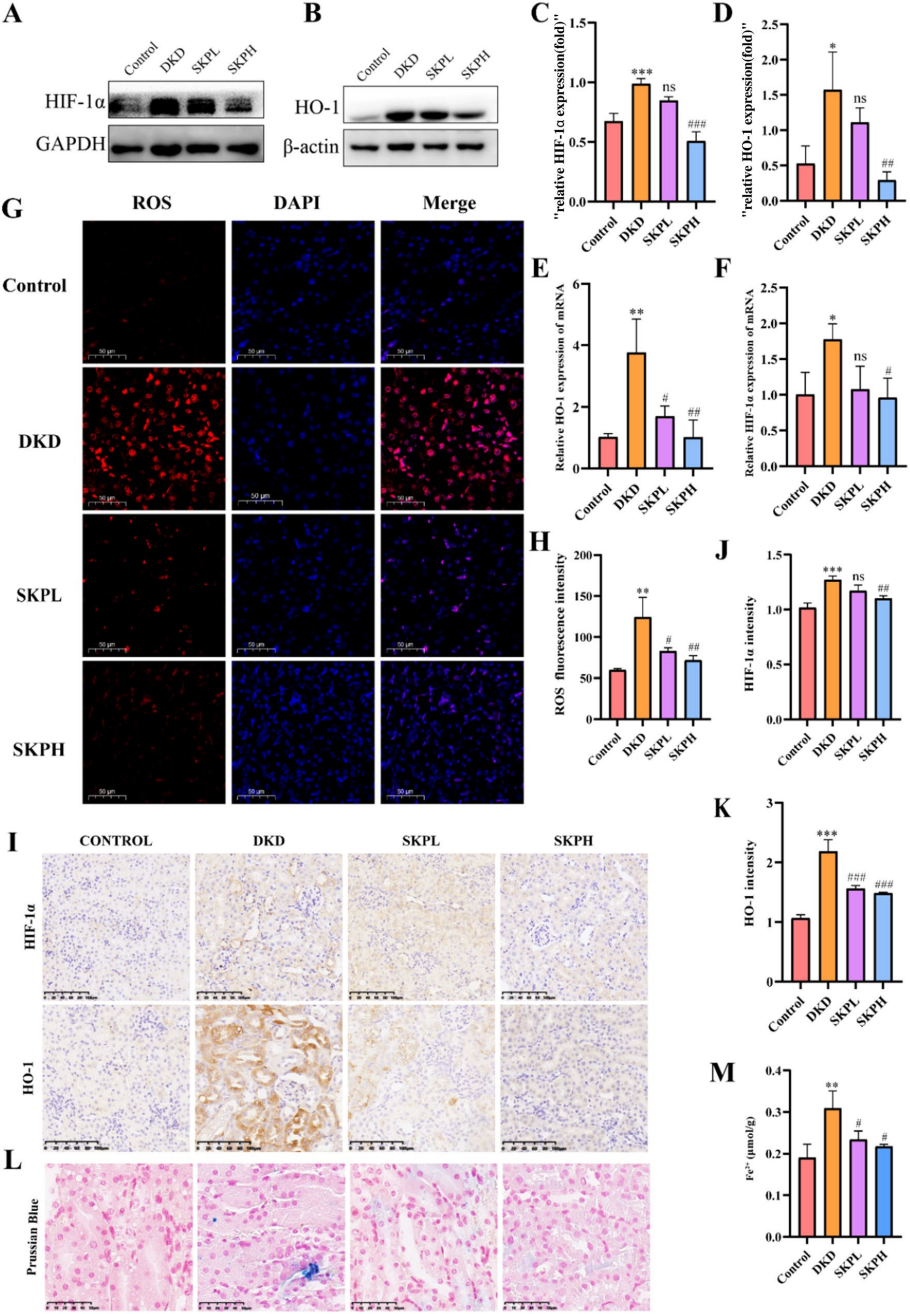
SKP inhibited the HIF-1α/HO-1 Signaling Pathway. **A**–**D** Western blot for HIF-1α and HO-1 in kidney tissues of each group and the quantitative analysis for Western blotting results of HIF-1α and HO-1. **E**, **F** The mRNA levels of HIF-1α and HO-1 in the renal tissues of each group. **G** Representative pictures of ROS changes in mice kidneys (bar = 50 µm). **H** Fluorescence intensity of ROS by image **J**. **I**–**K** Immunohistochemical staining assays of HIF-1α and HO-1 in kidneys and the quantifications of the expressions. (400 × magnification, bar = 100 µm). **L** Prussian blue staining of kidney tissues of mice. (800× magnification, bar = 50 µm). **M** Ferrous ion content in mice kidney tissues. *P < 0.05, **P < 0.01, and ***P < 0.001vs. control group. ^#^P < 0.05, ^##^P < 0.01, and ^###^P < 0.001 vs. DKD group, *ns* no significance

Based on network pharmacology, we hypothesized that SKP’s mechanism in treating DKD is closely linked to the HIF-1α/HO-1 signaling pathway. To assess the expression levels of key proteins in this pathway, Western blot analysis was conducted. The findings revealed that, in comparison to the model group, SKP treatment significantly reduced the protein expression levels of HIF-1α and HO-1 (Fig. 7A–D). These results were consistent with mRNA expression (Fig. 7E, F).

We investigated the ROS expression in mouse kidney tissues. In comparison to the control group, ROS levels were elevated in the model group, however, after treatment with SKP, a notable reduction in ROS production was observed (Fig. 7G, H). IHC demonstrated that SKP decreased HIF- 1α and HO-1 levels when compared to DKD mice (Fig. 7I–K). Prussian blue staining showed that ferroptosis occurred in renal tubules of DKD mice (Fig. 7L). As shown in Fig. 7M, ferrous ion content in DKD mice kidney tissues were obviously higher than that of normal group. These findings collectively suggest that SKP has the potential to alleviate renal damage induced by ferroptosis through the inhibition of HIF-1α/HO-1 signaling pathway.

## Discussion

The precise mechanism of SKP in DKD remains incompletely understood. Utilizing a DKD animal model induced by intraperitoneal injection of STZ combined with HFD. Many studies have shown that HFD combined with STZ is more likely to induce DKD [23, 24]. We evaluated renal function through crucial indicators such as 24h urine protein levels, urea nitrogen, and serum creatine. SKP exhibited significant improvement in renal injury in DKD mice, as evidenced by HE and PAS pathological staining, which revealed alleviation of renal tubular dilatation, vacuolar degeneration, and mild glomerular basement membrane thickening. Renal fibrosis is a major pathway in the progression of DKD, and may lead to ESRD. Excessive ROS production can trigger oxidative stress and accelerate fibrosis progression [25, 26]. Meanwhile, it has been demonstrated that ferroptosis may be associated with fibrosis [27]. We found that SKP could significantly reduce the expression of α-SMA, Fibronectin and Vimentin in renal tissues of DKD mice. This was also confirmed by Masson and Sirius Red staining.

In previous researches, we discovered that SKP can alleviate oxidative stress in DKD, as indicated by a marked reduction in MDA levels and an increase in the activity of antioxidant enzymes catalase and GSH [28]. Given the strong association between ferroptosis and oxidative stress, we build on our earlier experimental findings to explore the hypothesis that "SKP can attenuate ferroptosis in DKD". Our experiments have provided evidence supporting this hypothesis. Excessive intracellular ROS and iron trigger lipid peroxidation of phospholipids, leading to ferroptosis [29]. GPX4, an essential negative regulator of ferroptosis [30], catalyzes GSH to remove ROS, thereby safeguarding cell membrane integrity and reducing ferroptosis [31]. MDA, as the end product of lipid peroxidation, serves as an indicator of oxidative stress severity [29]. Upon examining these indicators, we found that SKP could effectively reduce ROS and MDA levels and increase the expression of GSH and GPX4 in kidney of DKD mice. In addition, the histochemical results of GPX4 suggested that the expression of GPX4 in the renal tubules of normal mice was higher than that of DKD mice, it was significantly improved after SKP treatment. Prussian blue staining showed that iron deposition occurred in the renal tubules of DKD mice compared with the normal group. All these findings validate our hypothesis on SKP’s impact on DKD, shedding lights on its potential as a therapeutic intervention.

By analyzing active ingredients and targets of SKP through network pharmacology, we screened out interaction targets of SKP with DKD and ferroptosis: TP53, IL6, EGFR, CTNNB1, ESR1, JUN, MYC, HIF1A, MYC, JUN, PTGS2, CASP8, CAV1, AR, ERBB2, GSK3B, MDM2, MAPK1, HSPA5, RELA, NFE2L2, HMOX1, HSPB1, RB1, GJA1, GSTP1, BIRC5, PRKCB, DPP4, PCNA, ALOX5, TRPV1, ATP5B, DPEP1. Furthermore, quercetin, kaempferol, isorhamnetin, beta-sitosterol, luteolin, stigmasterol and beta-carotene were identified as main active ingredients of SKP in the treatment of ferroptosis in DKD. Among them, quercetin can alleviate renal tubular cell death and inflammation in acute kidney injury by inhibiting ferroptosis [32]. Kaempferol is considered to have anti-inflammatory and anti-oxidation effects [33], which can inhibit ferroptosis of hepatocytes by activating Nrf2 pathway [34]. Isorhamnetin has anti-inflammatory and anti-cancer effects, and can also reduce MDA levels in the prefrontal cortex and hippocampus, thereby preventing oxidation [35]. Beta-sitosterol, which has a positive effect on anti-oxidation and anti-inflammation [36]. Luteolin can reduce renal anemia caused by renal fibrosis by regulating SIRT1/FOXO3 pathway [37]. Stigmasterol has been shown to reduce inflammation [38]. Additionally, beta-carotene has exhibited note-worthy anti-aging capabilities in both in vivo and in vitro studies [39]. KEGG enrichment analysis pinpointed the HIF-1 signaling pathway as a key player in SKP’s treatment of DKD-associated ferroptosis. Molecular docking heat map results further underscored robust binding activity between important targets and the main active components. Drawing from insights gained through the network pharmacology and molecular docking analysis, a compelling hypothesis emerges: SKP’s mitigation of ferroptosis in diabetic nephropathy primarily hinges on the inhibition of HIF-1α/HO-1 signaling pathway. This proposed mechanism sheds light on the intricate interplay between SKP’s active ingredients, molecular targets, and crucial pathways, offering a comprehensive perspective on its therapeutic potential in managing diabetic kidney disease.

**Fig. 8:**
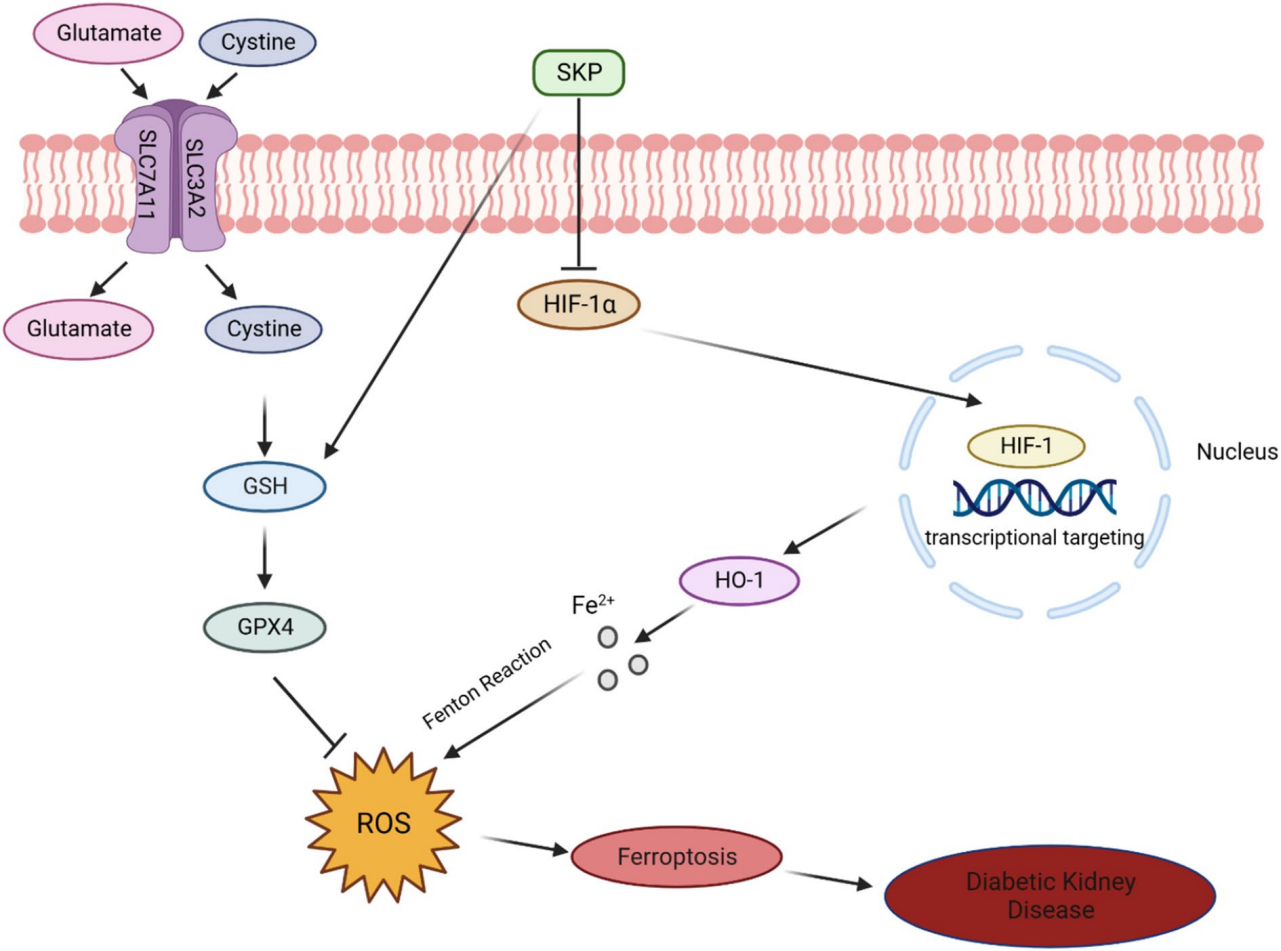
Research progress of SKP anti-ferroptosis ameliorating DKD

The regulatory factor Hypoxia-inducible factor-1 (HIF-1) plays a pivotal role in responding to Hypoxia, exsiting as a heterodimeric transcription factor comprising α subunit (HIF-1*α*) and *β* subunit (HIF-1*β*) [40, 41]. Under normoxia conditions, HIF-1α triggers ubiquitin-proteasome pathway to cause degradation [42]. However, in hypoxia environments, HIF-1α stabilizes, translocates into the nucleus, and forms a complex with Hif-1*β*. This complex binds to hypoxia-response element sites, regulating the expression of target genes [43, 44]. Heme Oxygenase-1 (HO-1) stands as a downstream target gene of HIF-1α [45]. It breaks down heme, generating metabolites like biliverdin, ferrous ion, carbon monoxide and biliverdin/bilirubin. Elevated HO-1 expression increases Fe^2+^ production, leading to Fenton reaction [46, 47], ROS overload and ultimately inducing ferroptosis [48] (Fig. 8). In our in vivo experiments, DKD mice exhibited significantly higher level of HIF-1α and HO-1 in their kidneys compared to normal mice. The observed reduction in ROS, HIF-1α and HO-1 expression with SKP suggests a potential attenuation of ferroptosis and alleviation of DKD through the inhibition of HIF-1α /HO-1 signaling.

While this study offers valuable insights into the mechanisms of SKP in DKD treatment, it is not without its limitations. The research relies predominantly on animal models, and its findings require further corroboration through human clinical trials. Additionally, future research should investigate the efficacy and long-term safety of SKP across various types and stages of DKD to fully establish its therapeutic potential.

##  Conclusion

In conclusion, SKP exhibits the potential to lower fasting blood glucose, enhance renal function, and mitigate renal fibrosis in DKD mice. Through network pharmacology analysis and in vivo experimental validation, we demonstrated for the first time that SKP may alleviate ferroptosis by inhibiting HIF-1*α*/HO-1 signaling, leading to a reduction in kidney damage, which is one of the mechanisms of SKP in the treatment of DKD.

### Supplementary Information

**Additional file 1: Table S1.** Specific information on compounds in The Herb-ingredient-targets gene network.

## Data Availability

All data for the duration of the study can be found in this article and appendix.

## Abbreviations

BP: Biological process
CC: Cellular component
DKD: Diabetic kidney disease
ESRD: End-stage renal disease
FBG: Fasting blood glucose
GO: Gene ontology
GPX4: Glutathione Peroxidase 4
GSH: Glutathione
HIF-1α: Hypoxia-inducible factor-1α
HO-1: Heme Oxygenase-1
KEGG: Kyoto Encyclopedia of Genes and Genomes
MDA: Malonaldehyde
MF: Molecular function
PPI: Protein–protein interaction
ROS: Reactive oxygen species
SKP: Shenkang pills
SKPH: SKP high-dose
SKPL: SKP low-dose
STZ: Streptozotocin
TCMSP: Traditional Chinese Medicine Systems Pharmacology
UniProt: Universal protein resource
VAL: Valsartan
