# Supplementary material for: SKP alleviates the ferroptosis in diabetic kidney disease through suppression of HIF-1α/HO-1 pathway based on network pharmacology analysis and experimental validation
Source: Chin Med. 2024 Feb 25;19:31. doi: 10.1186/s13020-024-00901-5 (PMC10894492; doi:10.1186/s13020-024-00901-5)
Supplement: Supplementary file 1 — Additional file 1: Table S1. Specific information on compounds in The Herb-ingredient-targets gene network. [file 13020_2024_901_MOESM1_ESM.pdf]

Table S1. Specific information on compounds in The Herb-ingredient-targets gene network.

Symbol	MOLID	Compounds
A1	MOL000211	Mairin
B1	MOL000354	isorhamnetin
C1	MOL000422	kaempferol
C2	MOL000098	quercetin
D1	MOL000449	Stigmasterol
D2	MOL000359	sitosterol
E1	MOL000073	ent-Epicatechin
F1	MOL001494	Mandenol
G1	MOL000358	beta-sitosterol
H1	MOL002773	beta-carotene
HQ1	MOL000239	Jaranol
HQ2	MOL000296	hederagenin
HQ3	MOL000033	(3S,8S,9S,10R,13R,14S,17R)-10,13-dimethyl-17-[(2R,5S)-5-propan-2yloctan-2-yl]-2,3,4,7,8,9,11,12,14,15,16,17-dodecahydro-1H-cyclopent a[a]phenanthren-3-ol
HQ4	MOL000371	3,9-di-O-methylnissolin

HQ5	MOL000378	7-O-methylisomucronulatol
HQ7	MOL000379	9,10-dimethoxypterocarpan-3-O-\u03b2-D-glucoside
HQ8	MOL000380	(6aR,11aR)-9,10-dimethoxy-6a,11a-dihydro-6H-benzofurano[3,2-c]chr omen-3-ol
HQ9	MOL000387	Bifendate
HQ10	MOL000392	formononetin
HQ11	MOL000417	Calycosin
HQ12	MOL000433	FA
HQ13	MOL000442	1,7-Dihydroxy-3,9-dimethoxy pterocarpene
JYZ1	MOL005030	gondoic acid
JYZ2	MOL008628	4'-Methyl-N-methylcoclaurine
YMC1	MOL001418	galeopsin
YMC2	MOL001420	ZINC04073977
YMC3	MOL001421	preleoheterin
YMC4	MOL001422	iso-preleoheterin
YMC5	MOL001439	arachidonic acid
YMX1	MOL013359	stigmasta-7-en-3-ol
YMX2	MOL001749	ZINC03860434
YMX3	MOL003044	Chryseriol

YMX4	MOL000006	luteolin
YMX5	MOL006756	Schottenol
DZ1	MOL002058	40957-99-1
DZ2	MOL004367	olivil
DZ3	MOL000443	Erythraline
DZ4	MOL006709	AIDS214634
DZ5	MOL007059	3-beta-Hydroxymethyllenetanshiquinone
DZ6	MOL007563	Yangambin
DZ7	MOL009009	(+)-medioresinol
DZ8	MOL009015	(-)-Tabernemontanine
DZ9	MOL009027	Cyclopamine
DZ10	MOL009029	Dehydrodiconiferyl alcohol 4,gamma'-di-O-beta-D-glucopyanoside_qt
DZ11	MOL009031	Cinchonan-9-al, 6'-methoxy-, (9R)-
DZ12	MOL009042	Helenalin
DZ13	MOL009047	(+)-Eudesmin
DZ14	MOL009053	4-[(2S,3R)-5-[(E)-3-hydroxyprop-1-enyl]-7-methoxy-3-methylol-2,3-di hydrobenzofuran-2-yl]-2-methoxy-phenol
DZ15	MOL009055	hirsutin_qt

DZ16	MOL009057	liriodendrin_qt
DZ18	MOL008240	(E)-3-[4-[(1R,2R)-2-hydroxy-2-(4-hydroxy-3-methoxy-phenyl)-1-meth ylol-ethoxy]-3-methoxy-phenyl]acrolein
DZ19	MOL011604	Syringetin
YXC1	MOL003851	Isoramanone
YXC2	MOL004350	Ruvoside_qt
YXC3	MOL004355	Spinasterol
